# A reliable and robust method for the upper thigh muscle quantification on computed tomography: toward a quantitative biomarker for sarcopenia

**DOI:** 10.1186/s12891-022-05032-2

**Published:** 2022-01-27

**Authors:** Yousun Ko, Youngbin Shin, Yu Sub Sung, Jiwoo Lee, Jei Hee Lee, Jai Keun Kim, Jisuk Park, Hye Sun Ko, Kyung Won Kim, Jimi Huh

**Affiliations:** 1 Biomedical Research Center, Asan Institute for Life Sciences, Asan Medical Center, Seoul, South Korea; 2 Clinical Research Center, Asan Medical Center, Seoul, South Korea; 3 Department of Convergence Medicine, University of Ulsan College of Medicine, Seoul, South Korea; 4 Department of Radiology and Research Institute of Radiology, Asan Medical Center, University of Ulsan College of Medicine, 88 Olympic-ro 43-gil, Songpa-gu, Seoul, South Korea; 5 Department of Radiology, Ajou University School of Medicine & Graduate School of Medicine, Ajou University Medical Center, 164 World cup-ro, Yeongtong-gu, Suwon, South Korea

**Keywords:** Sarcopenia, Computed tomography, Muscle, Biomarker, Landmark

## Abstract

**Background:**

We aimed to evaluate the feasibility of the upper thigh level as a landmark to measure muscle area for sarcopenia assessment on computed tomography (CT).

**Methods:**

In the 116 healthy subjects who performed CT scans covering from mid-abdomen to feet, the skeletal muscle area in the upper thigh level at the inferior tip of ischial tuberosity (SMA_UT_), the mid-thigh level (SMA_MT_), and L3 inferior endplate level (SMA_L3_) were measured by two independent readers. Pearson correlation coefficients between SMA_UT_, SMA_MT_, and SMA_L3_ were calculated. Inter-reader agreement between the two readers were evaluated using intraclass correlation coefficient (ICC) and Bland-Altman plots with 95% limit of agreement (LOA).

**Results:**

In readers 1 and 2, very high positive correlations were observed between SMA_UT_ and SMA_MT_ (*r* = 0.91 and 0.92, respectively) and between SMA_UT_ and SMA_L3_ (*r* = 0.90 and 0.91, respectively), while high positive correlation were observed between SMA_MT_ and SMA_L3_ (*r =* 0.87 and 0.87, respectively). Based on ICC values, the inter-reader agreement was the best in the SMA_UT_ (0.999), followed by the SMA_L3_ (0.990) and SMA_MT_ (0.956). The 95% LOAs in the Bland-Altman plots indicated that the inter-reader agreement of the SMA_UT_ (− 0.462 to 1.513) was the best, followed by the SMA_L3_ (− 9.949 to 7.636) and SMA_MT_ (− 12.105 to 14.605).

**Conclusion:**

Muscle area measurement at the upper thigh level correlates well with those with the mid-thigh and L3 inferior endpoint level and shows the highest inter-reader agreement. Thus, the upper thigh level might be an excellent landmark enabling SMA_UT_ as a reliable and robust biomarker for muscle area measurement for sarcopenia assessment.

**Supplementary Information:**

The online version contains supplementary material available at 10.1186/s12891-022-05032-2.

## Background

Sarcopenia is a muscle disease rooted in adverse muscle changes that accrue across a lifetime [1], and it has recently been assigned the International Classification of Disease (ICD-10CM) code [2]. Previous studies [1] have confirmed the association between sarcopenia and various adverse health outcomes, which indicates the importance of sarcopenia in health care. As the clinical significance of sarcopenia has been increasing, the appropriate techniques for evaluating skeletal muscle are gaining emphasis [3].

Cross-sectional imaging modalities such as computed tomography (CT) and magnetic resonance imaging (MRI) are considered to be the gold standard for non-invasive assessment of muscle quantity [4]. CT has been the most widely used because it is readily available in most hospitals worldwide.

A variety of landmarks, such as the third lumbar vertebral (L3) and mid-thigh, have been used for quantification of muscle area on CT [1, 5–8]. It has been reported that the skeletal muscle area measured at the level of the L3 correlates well with whole-body muscle mass [9]. Therefore, the L3 level on abdominopelvic CT has been recommended in the major international guidelines such as the European Working Group on Sarcopenia in Older People (EWGSOP) revised consensus guidelines updated in 2018 [5, 10]. Mid-thigh imaging has also been used in many research studies because it is a good predictor of appendicular muscle as well as whole-body skeletal muscle [11, 12]. However, the mid-thigh level might not be included in the routine abdominopelvic CT, requiring a dedicated CT scan for the thigh.

Recently, the importance of thigh muscle measurement has gained emphasis for sarcopenia assessment, because the thigh muscle showed strong relationship with physical activity and function in the elderly [13, 14]. In addition, the thigh muscles are the largest group of muscles in the body and the most important muscles for mobility. Thus, recent efforts to treat sarcopenia such as exercise therapy and electromyostimulation have emphasized to strengthen the thigh muscle mass and power [15].

The abdominopelvic CT is widely used as a standard diagnostic tool in many clinical settings for procedures such as cancer treatment, major surgery, and assessment of vascular disease [16, 17]. Thus, the body composition analysis in clinically acquired abdominopelvic CT scans is useful for patients who have required CT scans for their disease management [18]. However, it is difficult to measure the muscle area at mid-thigh in those patients. Instead, it is possible to measure the upper thigh muscle area on abdominopelvic CT.

The prerequisites to use the upper thigh level as a landmark for sarcopenia evaluation on CT are as follows: (1) the muscle area on the upper thigh level should be correlated well with that on the mid-thigh level and the L3 level and (2) the muscle area measurement on the upper thigh level should be reliable based on a robust anatomic landmark. In this study, we aim to evaluate whether or not these prerequisites are satisfied or not to use the upper thigh level as a landmark on CT.

## Methods

### Study subjects

The institutional review board of two participating institutions (Ajou University Hospital, Asan Medical Center) approved this retrospective study and waived the requirement for informed consent for the use of patient data.

In both institutions, each institutional computerized data warehouse was searched to find healthy subjects who underwent CT scans with scan coverage of both abdomen and thigh. In both institutions, lower extremity CT was scanned from mid-abdomen to feet, mainly to evaluate for extremity pain, varicose vein, and varicocele. We selected patients consecutively with the following inclusion criteria: (1) adult patients > 19-year-old, (2) who underwent lower extremity CT from January 2020 to July 2020, and (3) patients without significant underlying disease in the abdomen and extremity, except for varicose vein and varicocele. We excluded those who were in the disease states such as vascular thrombosis, fracture, malignant tumors, edema, muscle degeneration, or atrophy. We recorded demographic information such as age, sex, height, weight, and reason for CT scanning. Body mass index (BMI) was calculated as the weight in kilograms divided by the height squared in meters (kg/m^2^).

### CT imaging

In Ajou University Hospital, CT scans were performed with two multidetector CT scanners (Revolution EVO, GE Medical Systems, Milwaukee, WI; Aquilion ONE; Canon Medical Systems, Tokyo, Japan) using the following scanning parameters: tube voltage 100 kVp; automatic exposure control; matrix 512 × 512; and slice thickness 5 mm. In Asan Medical Center, CT scans in this study were performed with a multi-detector CT scanner (SOMATOM definition plus, Siemens, Erlangen, Germany) with the following protocols: tube voltage 120 kVp; tube current 200 mA; matrix 512 × 512; and slice thickness 3 mm. All images were obtained after the intravenous administration of nonionic iodinated contrast agent at a rate of 3–4 mL/s. In this study, we used the axial CT venography images, which were scanned at 3 min after contrast injection.

### Level of muscle measurement

**Fig. 1:**
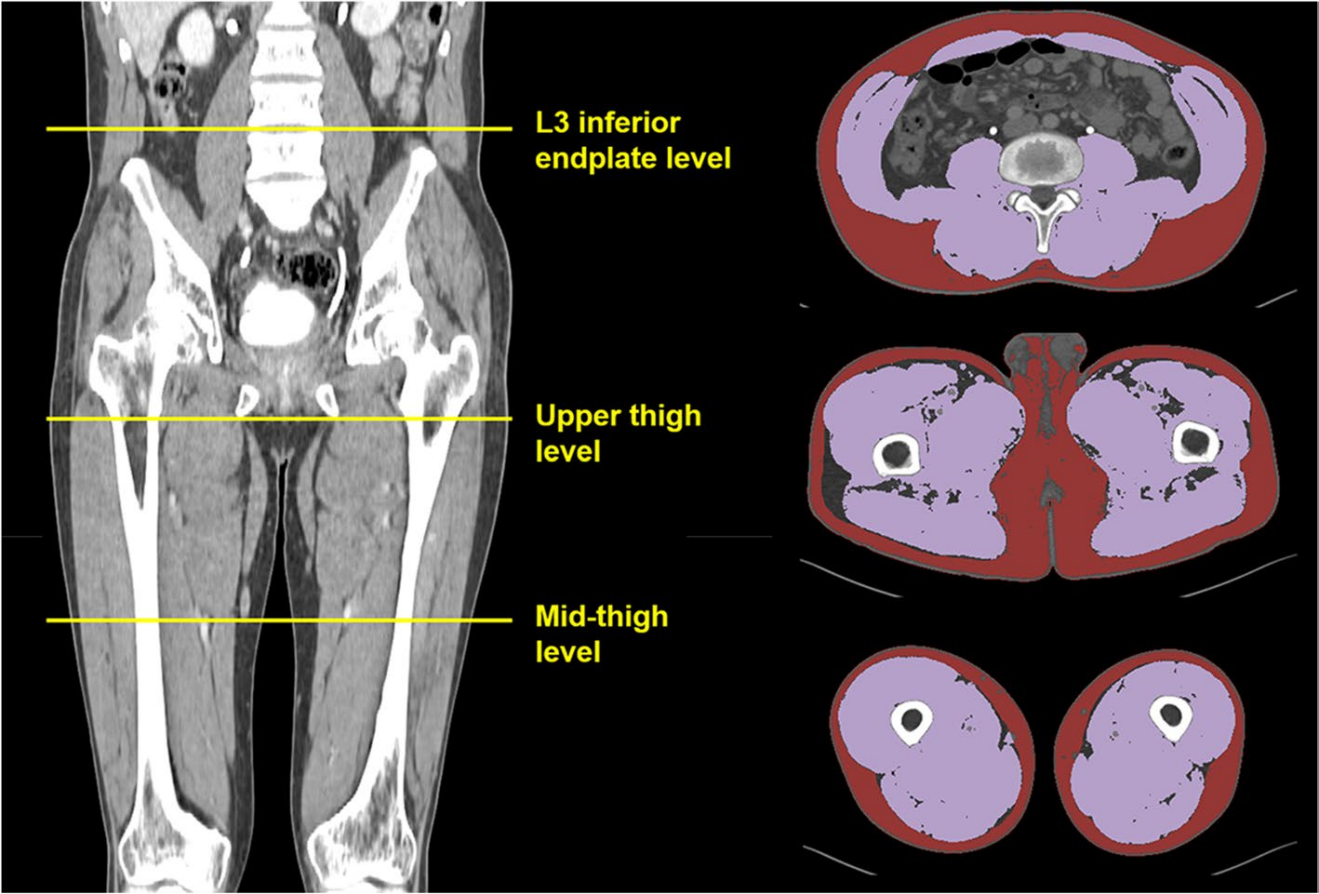
Measurement levels of body composition

The level of muscle measurement was illustrated in Fig. 1. We defined the level of the upper thigh as the inferior tip of the ischial tuberosity, where the lowest part of the ischial bone was demonstrated on CT images. The level of the mid-thigh was defined as the middle distance between the distal end of the femoral neck and the first slice showing the intercondylar fossa [19]. The level of L3 was defined as the inferior endplate of the L3 [10]. Our study coordinator provided the method to select levels of muscle measurement to image analysis teams. For the mid-thigh level selection, we recommended to use coronal reconstructed images to choose a midpoint of the femur.

### Body morphometric analysis

We organized two image analysis teams, as each team included an abdominal radiologist and an image analyst. Two radiologist readers (J.H. with 11 years of experience, J.K.K. with 20 years of experience) independently selected axial images from the levels of the L3, upper thigh, and mid-thigh, respectively. Then, two image analysts (J.P. with 7 years of experience, H.K. with 9 years of experience) independently created segmentation maps of skeletal muscle and subcutaneous fat using semi-automated segmentation software (AsanJ-Morphometry™, Asan Image Metrics, Seoul, Korea, http://datasharing.aim-aicro.com/morphometry) on the selected axial images [5]. All segmentation maps were reviewed and corrected as necessary by two abdominal radiologists independently.

The skeletal muscle area (SMA) was demarcated using a predetermined threshold (− 29 to + 150 Hounsfield unit [HU]). Here, the subcutaneous fat area (SFA) was also demarcated using a fat tissue threshold (− 190 to − 30 HU) [20]. The cross-sectional area (cm^2^) of the segmented regions were obtained. At upper and mid-thigh levels, we combined measured areas in the right thigh and left thigh.

## Statistical analysis

Data are expressed as mean ± standard deviation (SD). The mean values of measured muscle areas at the L3, upper thigh, and mid-thigh levels of two readers were demonstrated using Box-whisker plots and compared by analysis of variance (ANOVA) and *post-hoc* multiple comparison tests.

Correlation analysis was performed to describe the strength (degree) and the direction of the relationship between variables using Pearson’s correlation coefficient. The strength of correlation was categorized according to *r* value as very high (≥ 0.90), high (0.70 to 0.89), moderate (0.50 to 0.69), low (0.30 to 0.49), and negligible (0.00 to 0.29) [21].

The measurement agreements and between readers 1 and 2 (inter-reader agreement) were assessed on the basis of the intraclass correlation coefficient (ICC) of a single measurement calculated according to the two-way random-effects model for absolute agreement. Bland-Altman plots were also constructed with the mean difference and 95% limit of agreement (LOA).

Statistical significance was considered when the *p*-value was < 0.05. Data were analyzed using MedCalc version 13.1.2 (MedCalc Software, Ostend, Belgium).

## Results

### Patient demographics

The average age (mean ± SD) of the 116 subjects was 53.4 ± 12.5 years (range, 22–80 years; median, 55; interquartile range, 46–62). The subjects included 85 men (mean age, 50.4 years; range, 29–75 years) and 31 women (mean age, 54.5 years; range, 22–80 years) (Table 1).

**Table 1:** Patient characteristics

Characteristics	Data (***n*** = 116)
Age (years)	53.4 ± 12.5
Sex ratio (M:F)	85:31
Weight (kg)	67.8 ± 12.4
Height (cm)	166.7 ± 7.7
Body mass index (kg/m^2^)	24.3 ± 3.6
Reason of CT scanning	
Evaluation for extremity pain	16 (13.8)
Varicose vein	81 (69.8)
Varicocele	19 (16.4)

*Note*: Data are mean ± standard deviation or numbers (and percentages)

### Comparison of muscle areas between measurement levels

**Fig. 2:**
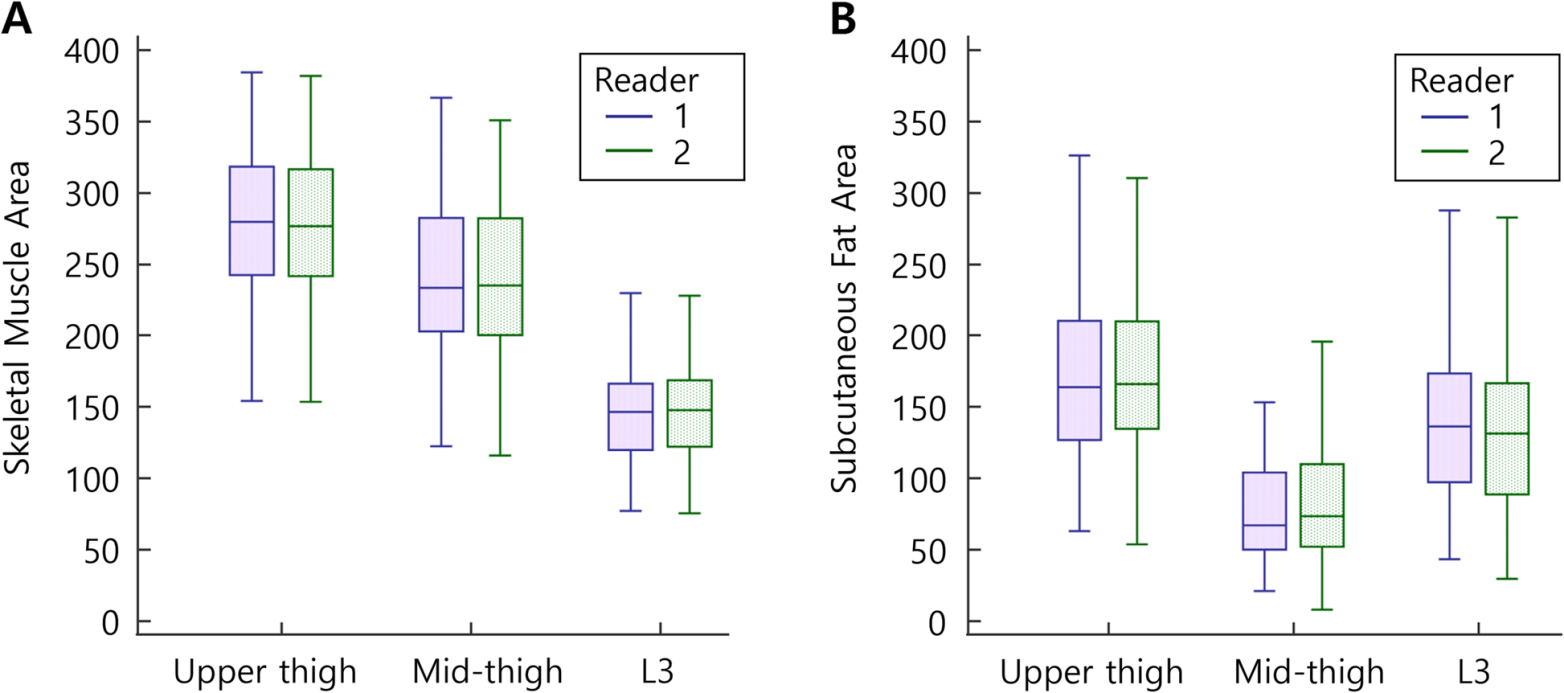
The cross-sectional areas between measurement levels in each reader (A) skeletal muscle area, (B) subcutaneous fat area

The measured cross-sectional areas (cm^2^) of the SMA and SFA values at the L3 (SMA_L3_, SFA_L3_), upper thigh (SMA_UT_, SFA_UT_), and mid-thigh (SMA_MT_, SFA_MT_) levels are presented in Fig. 2. In readers 1 and 2, the SMA_UT_ showed the highest values (279.5 ± 53.0 and 277.3 ± 53.1, respectively), followed by the SMA_MT_ (241.4 ± 56.4 and 238.4 ± 55.7, respectively) and SMA_L3_ (145.5 ± 33.0 and 147.5 ± 34.5, respectively), with significance differences between pairs (*p* <  0.001 for ANOVA; *p* <  0.001 for all pair-wise comparisons).

In readers 1 and 2, the SFA_UT_ showed the highest values (173.9 ± 62.1 and 177.2 ± 62.1, respectively), followed by the SFA_L3_ (146.2 ± 63.6 and 137.9 ± 63.4, respectively) and SFA_MT_ (82.3 ± 45.5 and 87.2 ± 48.2, respectively), with significance differences between pairs (*p* <  0.001 for ANOVA; *p* <  0.05 for all pair-wise comparisons).

### Correlation of muscle and fat areas between measurement levels

In readers 1 and 2, very high positive correlations were observed between SMA_UT_ and SMA_MT_ (*r* = 0.91 and 0.92, respectively) and between SMA_UT_ and SMA_L3_ (*r* = 0.90 and 0.91, respectively), while high positive correlation were observed between SMA_MT_ and SMA_L3_ (*r =* 0.87 and 0.87, respectively) (Table 2).

**Table 2:** Pearson correlation coefficients of muscle and fat areas between measurement levels

Correlation		Reader 1		Reader 2	
		Coefficient ***r***	***p-***value	Coefficient ***r***	***p-***value
SMA	b/w Upper thigh and Mid-thigh	0.91	< 0.001	0.92	< 0.001
	b/w Upper thigh and L3	0.90	< 0.001	0.91	< 0.001
	b/w Mid-thigh and L3	0.87	< 0.001	0.87	< 0.001
SFA	b/w Upper thigh and Mid-thigh	0.90	< 0.001	0.92	< 0.001
	b/w Upper thigh and L3	0.73	< 0.001	0.73	< 0.001
	b/w Mid-thigh and L3	0.68	< 0.001	0.68	< 0.001

*Abbreviations*: *SMA* skeletal muscle area, *SFA* subcutaneous fat area, *b/w* between

Regarding the fat areas, in readers 1 and 2, we observed very high correlation between SFA_UT_ and SFA_MT_ (*r* = 0.90 and 0.92, respectively), high correlation between SFA_UT_ and SFA_L3_ (*r* = 0.73 and 0.73, respectively), and moderate correlation between SFA_MT_ and SFA_L3_ (*r* = 0.68 and 0.68, respectively) (Table 2).

### Reliability of muscle area measurement

Based on ICC values, the inter-reader agreement was the best in the SMA_UT_ (0.999), followed by the SMA_L3_ (0.990) and SMA_MT_ (0.956). The mean difference and 95% LOAs in the Bland-Altman plots also indicated that the inter-reader agreement of the SMA_UT_ (0.526 cm^2^; − 0.462 to 1.513) was the best, followed by the SMA_L3_ (− 1.156; − 9.949 to 7.636) and SMA_MT_ (1.250; − 12.105 to 14.605) (Table 3). The Bland-Altman plots were presented in Supplementary Fig. 1.

**Table 3:** Inter-reader agreement between readers 1 and 2

Agreement		Bland-Altman Analysis		Intraclass correlation coefficient
		Mean of differences (cm^**2**^)	95% LOA (cm^**2**^)	
SMA	Upper Thigh	0.526	−0.462 to 1.513	0.999 (0.9933 to 0.9997)
	Mid-Thigh	1.250	−12.105 to 14.605	0.956 (0.9373 to 0.9697)
	L3	−1.156	−9.949 to 7.636	0.990 (0.9853 to 0.9933)
SFA	Upper Thigh	−2.581	−14.206 to 9.044	0.985 (0.9745 to 0.9909)
	Mid-Thigh	−5.223	− 44.335 to 33.889	0.952 (0.9223 to 0.9687)
	L3	5.767	−15.919 to 27.453	0.981 (0.9538 to 0.9901)

*Abbreviations*: *SMA* skeletal muscle area, *SFA* subcutaneous fat area, *LOA* limit of agreement

When we explored the reason why the inter-reader agreement of SMA_UT_ was the best, we found that the CT slices selected for SMA_UT_ differed in 10 patients (8.62%) between readers 1 and 2, while those differed in 26 patients (22.4%) for SMA_L3_, and in 90 patients (77.6%) for SMA_MT_. As presented in Supplementary Fig. 2, the differences in selecting measurement level between readers 1 and 2 were as follows: upper thigh level (mean 0.06 ± 0.25 cm, median 0.0 cm, interquartile range 0.0–0.0 cm), mid-thigh level (mean 0.66 ± 0.74 cm, median 0.5 cm, interquartile range 0.25–0.75 cm), and L3 inferior endplate level (mean 0.52 ± 0.57 cm, median 0.5 cm, interquartile range 0.5–0.5 cm).

The difference in the L3 level CT slice between readers 1 and 2 was mainly attributed to a difficulty in identifying the L3 level in patients with thoracolumbar variations (*n* = 4), lumbosacral variations (*n* = 7), and a slight difference in identifying the exact L3 inferior endplate level (*n* = 15). In contrast, the difference in the CT slice of mid-thigh level between readers 1 and 2 was mainly attributed to the lack of an exact anatomic landmark. Indeed, readers had difficulty in estimating the middle distance between the distal end of the femoral neck and the first slice showing the intercondylar fossa on axial images (*n* = 90).

## Discussion

Our study demonstrated that skeletal muscle measurement in the upper thigh level at the inferior tip of ischial tuberosity correlated well with those in the mid-thigh level and L3 inferior endplate level. The muscle measurement in the upper thigh level showed the highest inter-reader agreement compared to those in the mid-thigh and L3 level, because two readers selected identical CT slices for the upper thigh level in most patients (106 out of 116). In contrast, the numbers of identical CT slices between readers were 26 for the mid-thigh level and 90 for the L3 level. Thus, it might be feasible to use the upper thigh level on abdominal CT as an anatomic landmark enabling SMA_UT_ as a reliable and robust biomarker for muscle area measurement for sarcopenia assessment.

The strength of our study is that this is the first study to evaluate feasibility of the upper thigh muscle measurement on CT as a biomarker for sarcopenia assessment. We also proposed the detailed landmark for the upper thigh muscle area measurement on CT. It is worth discussing advantages and drawbacks of using the upper thigh level on abdominal CT as a landmark for sarcopenia assessment. First of all, the muscle area measurement at the upper thigh level can reflect the whole body muscle, as SMA_UT_ showed a very high correlation with SMA_MT_ as well as SMA_L3_. Prior studies demonstrated that the skeletal muscle measured at the L3 level on abdominal CT could represent the whole-body muscle [5, 9, 22, 23]. In addition, it has been known that skeletal muscle measured in the mid-thigh level on extremity CT can be a good predictor of whole-body skeletal muscle [11]. The muscles in the L3 level reflect well the axial muscles while the muscles in the mid-thigh level reflect well the appendicular muscles. Thus, there might be a discrepancy between axial muscles and appendicular muscles for sarcopenia assessment. Indeed, our study showed that the correlation between SMA_MT_ and SMA_L3_ was the lowest (*r*, 0.87) compared with those between SMA_UT_ and SMA_MT_ (*r,* from 0.91 to 0.92) and between SMA_UT_ and SMA_L3_ (*r*, from 0.90 to 0.91). The upper thigh muscles might represent both axial muscles and appendicular muscles because the upper thigh level is the transition point between pelvic girdle (i.e., axial skeletons) and lower extremities (i.e., appendicular skeletons).

Second, the upper thigh level, defined as the inferior tip of the ischial tuberosity, can be easily identified on CT, because it is the lowest slice where the ischial bone was demonstrated on CT images. In contrast, readers might have difficulty in determining the exact mid-thigh level on axial CT images because the definition of mid-thigh, the middle distance between the distal end of the femoral neck and the first slice showing the intercondylar fossa, does not use exact anatomic landmark to pick mid-thigh level. Readers had to use coronal reconstructed images to identify the mid-point between the femoral neck and the intercondylar fossa, which required additional steps and measurement time. Furthermore, previous studies [11, 19, 24, 25] using the mid-thigh as an anatomic landmark adopted various definitions of mid-thigh level, which may hamper consistent muscle measurement.

As for the vertebral variations which might affect the L3 level selection, there were a total of 19 patients with variations in our study, as follows: thoracolumbar variation (*n* = 6), lumbosacral variation (*n* = 10), numeric variation (n = 1), and combined variation (*n* = 2). Of these 19 patients, disagreement of L3 level between readers 1 and 2 was performed in 11 patients.

In general, abdominopelvic CT is widely used to evaluate various diseases and is readily available around the world. In most abdominopelvic CT, the upper thigh level is covered, while the mid-thigh CT requires an additional CT scan for the extremity. Thus, if we adopt the upper thigh level as an anatomic landmark as well as the L3 level, we can acquire muscle area measurement for both axial and appendicular muscles in most patients with clinically acquired abdominopelvic CT.

In our study, we used two different CT protocols between two institutions. The Ajou University Hospital used 100 kVp, automatic exposure control, and 5 mm slice thickness, whereas the Asan Medical Center used 120 kVp, 200 mA, and 3 mm slice thickness. This may raise an issue of measurement variability across institutions. According to a recent paper which used a body morphometry phantom to evaluate the effects of CT protocols on muscle area measurement, the skeletal muscle area (threshold, − 29 to 150 HU) was constant, regardless of the CT protocols (tube voltage, tube current, slice thickness and the image reconstruction algorithm). The SNR decreased with low tube voltage, low tube current, and in sections with thin slices, whereas the SNR did not affect on the measurement value of muscle mass [26].

To our best knowledge, this is the first study to propose to use the upper thigh level as a landmark for muscle measurement. However, there are some limitations. First, this study did not demonstrate the association between the measured upper thigh muscle area and the whole-body muscle mass or muscle function, warranting future study. Second, the subjects enrolled in this study were health patients, which might limit the generalizability of the study results. The measurement of muscle and fat tissues on CT may show more variation in the diseased or truly sedentary older subjects than healthy subjects. This is the first study to evaluate the feasibility of the upper thigh muscle measurement as a quantitative biomarker, thus we started research from healthy subjects. Our future study would be to validate the upper thigh muscle measurement as a biomarker for sarcopenia in patients with various diseases.

## Conclusions

Muscle area measurement at the upper thigh level correlates well with those with the mid-thigh and L3 inferior endplate level and shows the highest inter-reader agreement. Thus, the upper thigh level might be an excellent landmark enabling SMA_UT_ as a reliable and robust biomarker for muscle area measurement for sarcopenia assessment.

## Supplementary Information

**Additional file 1 Supplementary Fig. 1**. Bland-Altman plots to evaluate inter-reader agreement between readers 1 and 2 for measurement of the skeletal muscle area (SMA) and the subcutaneous fat area (SFA).

**Additional file 2 Supplementary Fig. 2**. Box-whisker plots for the difference in selecting measurement level between readers 1 and 2.

## Data Availability

The datasets used and/or analyzed during the current study are available from the corresponding author on reasonable request.

## Abbreviations

ANOVA: Analysis of variance
BMI: Body mass index
CT: Computed tomography
EWGSOP: European Working Group on Sarcopenia in Older People
HU: Hounsfield unit
ICC: Intraclass correlation coefficient
ICD: International Classification of Disease
L3: Third lumbar vertebral
LOA: Limit of agreement
MRI: Magnetic resonance imaging
SD: Standard deviation
SFA: Subcutaneous fat area
SMA: Skeletal muscle area
