# Supplementary material for: A reliable and robust method for the upper thigh muscle quantification on computed tomography: toward a quantitative biomarker for sarcopenia
Source: BMC Musculoskelet Disord. 2022 Jan 27;23:93. doi: 10.1186/s12891-022-05032-2 (PMC8796642; doi:10.1186/s12891-022-05032-2)

**Supplementary Figure 1**. Bland-Altman plots to evaluate inter-reader agreement between readers 1 and 2 for measurement of the skeletal muscle area (SMA) and the subcutaneous fat area (SFA).

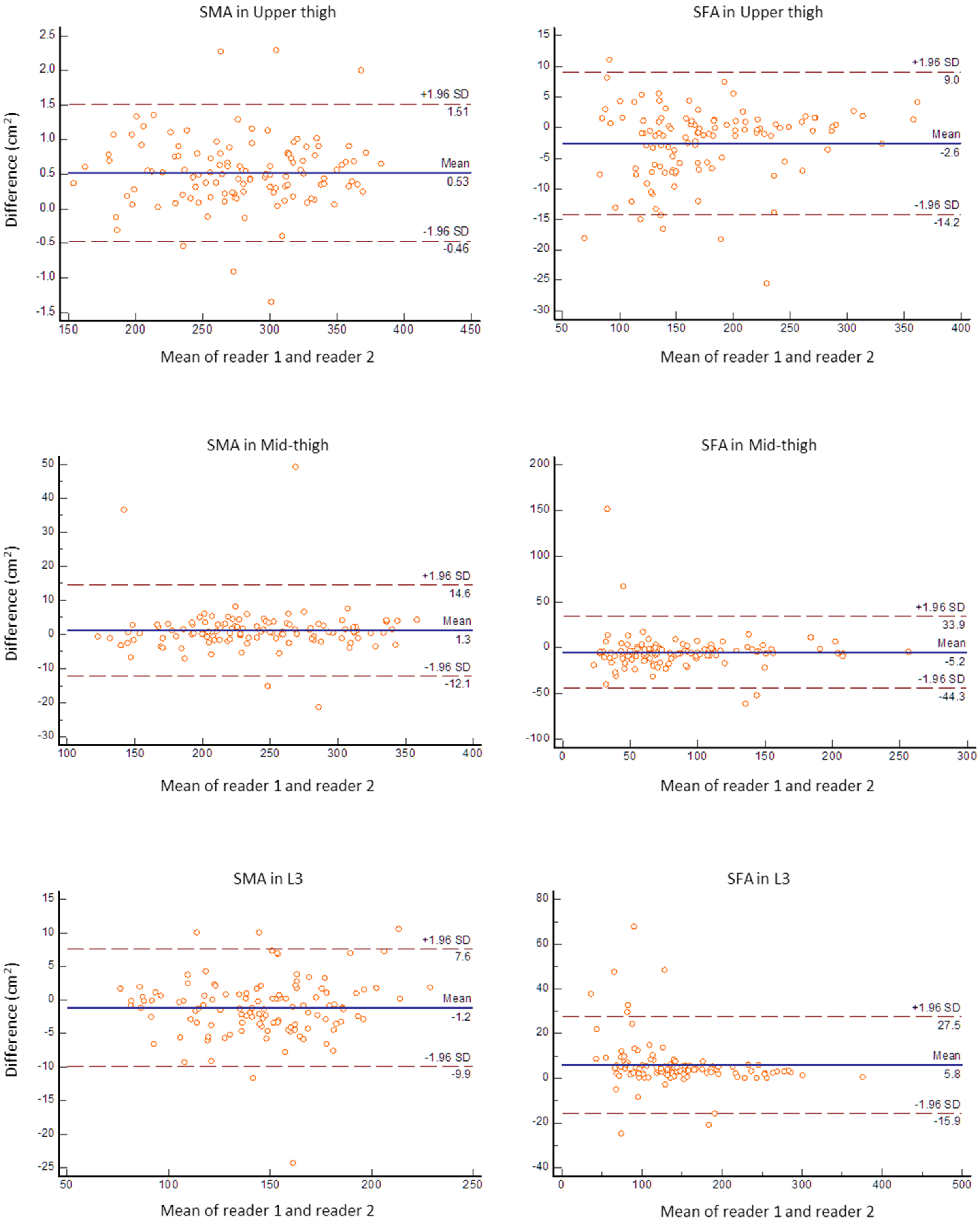

Supplement: Supplementary file 1 — Additional file 1 Supplementary Fig. 1. Bland-Altman plots to evaluate inter-reader agreement between readers 1 and 2 for measurement of the skeletal muscle area (SMA) and the subcutaneous fat area (SFA). [file 12891_2022_5032_MOESM1_ESM.pdf]
