# Supplementary material for: A reliable and robust method for the upper thigh muscle quantification on computed tomography: toward a quantitative biomarker for sarcopenia
Source: BMC Musculoskelet Disord. 2022 Jan 27;23:93. doi: 10.1186/s12891-022-05032-2 (PMC8796642; doi:10.1186/s12891-022-05032-2)

Supplementary Figure 2. Box-whisker plots for the difference in selecting measurement level between readers 1 and 2.

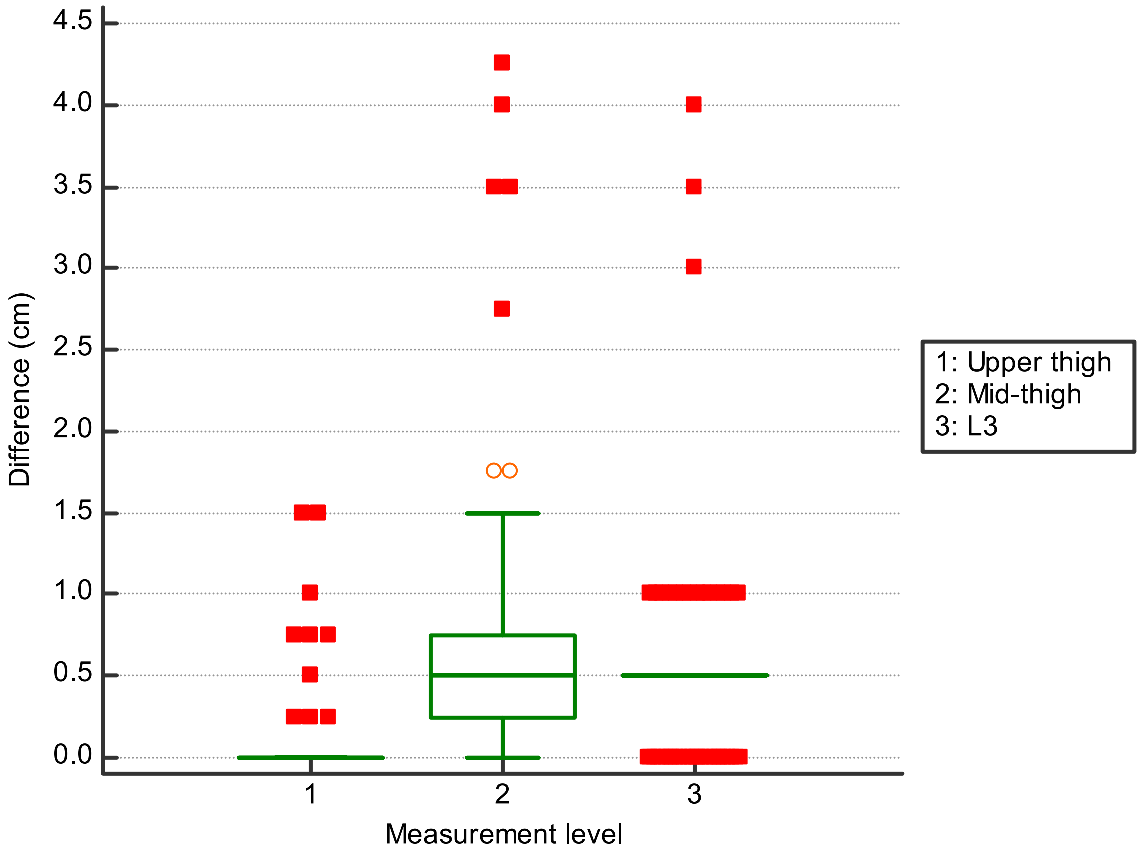

Supplement: Supplementary file 2 — Additional file 2 Supplementary Fig. 2. Box-whisker plots for the difference in selecting measurement level between readers 1 and 2. [file 12891_2022_5032_MOESM2_ESM.pdf]
